# Comparison of three tests of homogeneity of odds ratios in multicenter trials with unequal sample sizes within and among centers

**DOI:** 10.1186/1471-2288-11-58

**Published:** 2011-04-26

**Authors:** Zahra Bagheri, Seyyed Mohammad Taghi Ayatollahi, Peyman Jafari

**Affiliations:** 1 Department of Biostatistics, Shiraz University of Medical Sciences, Shiraz, Iran

## Abstract

**Background:**

Mixed effects logistic models have become a popular method for analyzing multicenter clinical trials with binomial data. However, the statistical properties of these models for testing homogeneity of odds ratios under various conditions, such as within-center and among-centers inequality, are still unknown and not yet compared with those of commonly used tests of homogeneity.

**Methods:**

We evaluated the effect of within-center and among-centers inequality on the empirical power and type I error rate of the three homogeneity tests of odds ratios including likelihood ratio (LR) test of a mixed logistic model, DerSimonian-Laird (DL) statistic and Breslow-Day (BD) test by simulation study. Moreover, the impacts of number of centers (*K*), number of observations in each center and amount of heterogeneity were investigated by simulation.

**Results:**

As compared with the equal sample size design, the power of the three tests of homogeneity will decrease if the same total sample size, which can be allocated equally within one center or among centers, is allocated unequally. The average reduction in the power of these tests was up to 11% and 16% for within-center and among-centers inequality, respectively. Moreover, in this situation, the ranking of the power of the homogeneity tests was BD≥DL≥LR and the power of these tests increased with increasing *K*.

**Conclusions:**

This study shows that the adverse effect of among-centers inequality on the power of the homogeneity tests was stronger than that of within-center inequality. However, the financial limitations make the use of unequal sample size designs inevitable in multicenter trials. Moreover, although the power of the BD is higher than that of the LR when *K*≤6, the proposed mixed logistic model is recommended when *K*≥8 due to its practical advantages.

## Background

The results from multicenter clinical trials or meta-analysis studies with binomial data are often summarized in *K *2 × 2 contingency tables, where *K *denotes the total number of centers or studies. Combining data in such tables and proposition a summary measure is the primary objective of such studies. However, before computing the overall odds ratio, we often need to assess whether the specific odds ratios are homogeneous across tables [1-4].

Nowadays, investigators have a wide range of methods available for this purpose, including model-based and test-based approaches. The excellent simulation studies conducted by pioneer researchers in this field assist us in choosing the most appropriate test for the assessment of homogeneity among *K *2 × 2 tables [1-6]. Nevertheless, the results of these simulation studies indicate that homogeneity tests show different behaviors under combinations of parameters such as the number of centers, center sizes and amount of heterogeneity [3,5,7].

In recent years, a class of models called mixed logistic models has been used for analysis of multicenter clinical trials with binomial data. Although Agresti has discussed a likelihood ratio (LR) test based on a mixed logistic model for testing homogeneity of odds ratios in *K *2 × 2 contingency tables [8], the statistical properties of this test and the other traditional homogeneity tests such as Breslow-Day (BD) [9] and DerSimonian-Laird (DL) [10] are still unknown. A situation which occurs frequently in multicenter trials and has not been evaluated in previous studies is the effect of unequal sample size designs on the statistical properties of these homogeneity tests. For example, in some multicenter clinical trials, when one center is larger, it may seem reasonable to select a larger sample from it, but this leads to among-centers inequality. Moreover, within-center inequality could occur when the costs of two treatment groups are very different. In this situation, due to financial restrictions, it is reasonable to allocate more patients to the cheaper treatment in each center. This simulation study hence compares the empirical power and type I error rate of the three tests of homogeneity of odds ratios, including LR, DL and BD tests when the sample size is unequal within one center or among centers.

## Methods

Consider a series of *K *independent 2 × 2 contingency tables, with the data in the *k*th table denoted as shown in Table 1. In this paper, *K *is the number of centers in multicenter clinical trials.

**Table 1:** Summary of data from the *k*th 2 × 2 contingency table

	Success	Failure	Total
Treatment 1 (*x*_1*k*_)	*y*_1*k*_	*n*_1*k *_- *y*_1*k*_	*n*_1*k*_
Treatment 2 (*x*_2*k*_)	*y_2k_*	*n*_2*k *_- *y*_2*k*_	*n*_2*k*_
Total	*t_k_*	*t_k _- n_k_*	*n_k_*

Suppose that each *y_ik _*follows a binomial distribution with parameters *n_ik _*and *π_ik _*(i = 1,2; k = 1,2,...,K). Let *n_ik _*denote the total number of observations in the *i*th treatment arm and *k*th center, and let *π_ik _*denote the success probability at treatment level *x_ik _*in the *k*th center, where *x_ik _*is the treatment indicator with *x_ik _*= 1 representing treatment 1 and *x_ik _*= 0 treatment 2.

In this paper, we focus on the following logistic-normal probability model in order to assess the homogeneity of odds ratios:(1)

In this model, the segment *α + βx_ik _*is the fixed effect part in which *β *is the common treatment effect. Here, *u_k _*and *b_ik _*are independent random components of the model, where *u_k _*is the center effect and  and the parameter  summarizes center heterogeneity. In addition, *b_ik _*is the center-by-treatment interaction random effect,  and the parameter  describes variability in the log-odds ratios [8]. The two advantages of working with this model are: first, a test of homogeneity of odds ratios can be performed by testing the null hypothesis:  against ; and, second, the common treatment effect and also center-specific odds ratios can be obtained by estimating *β *and *b_ik _*[8].

It should be noted that the homogeneity test can be performed using likelihood ratio (LR) test Δ = -2(*l_0 _*- *l_1_*), where *l_0 _*is the log-likelihood under the assumption of homogeneity of odds ratios and when there is just one random effect, *u_k_*, in the Model 1 and *l_1 _*is the log-likelihood when both *u_k _*and *b_ik _*are in the model. Under the null hypothesis, the asymptotic distribution of the LR test is a mixture of a chi-squared distribution with zero and one degrees of freedom, respectively, both with weight of 1/2 [11,12].

Since the properties of the LR test in order to assess homogeneity of odds ratios had not been evaluated in the previous studies, we investigated the behavior of this test under various conditions, particularly under within-center and among-centers inequality in multicenter trials and also compared it with the other two most common test statistics, including DL and BD tests. Brief calculation details of these statistics are given in the appendix [9-12].

### Simulation study

We studied the statistical properties of the three mentioned-above homogeneity tests, in terms of empirical power and type I error rate, by simulation in SAS statistical package v 9.1. Our simulation design was based on the mixed logistic model 1; we set the fixed effect parameters for all simulation scenarios arbitrarily as: *α = -1 *and *β = 1 *. The random components *u_k _*and *b_ik _*are generated independently from normal distribution with mean zero and variance  and . In order to generate the response variable, the binomial distribution with parameters *n_ik _*and  was used. The influence of within-center and among-centers inequality, different values of *K*, ,  and also number of observations in each centers, *n_k_*, on the statistical properties of all the homogeneity tests were evaluated. We generated 1000 random data sets for each simulation scenario. Namely, we chose three values for *K *(4, 6 and 8), two values for  (0.1 and 0.5) and five values for  (0, 0.2, 0.4, 0.6 and 0.8). In the equal sample size design, in which equal numbers of patients were allocated between two treatment arms and also among all centers, the number of individuals per center, *n_k_*, was set at 40, 100 and 200. In addition, in this study two forms of unequal sample size designs were considered. In the first one, ie within-center inequality, the same sample size, which can be allocated equally between two treatment arms within a center, is allocated unequally in the ratio of 3:1. In this case, the sample sizes were considered equal in all centers. In the latter one, ie among-centers inequality, the same total sample size, which can be allocated equally among centers, is allocated unequally. In this situation, one center has a much larger sample size in comparison with the other ones. The exact details of different sample size configurations are described in Table 2. In addition, SAS simulation code for performing the three homogeneity tests is presented in the additional file 1.

**Table 2:** Description of different configurations of sample size in equal and unequal sample size designs.

K	Sample size per treatment arm
	**Equal sample size design:** **E_1_**: n_tot_= 160(20:20, 20:20, 20:20, 20:20) **E_2_**: n_tot_= 400(50:50, 50:50, 50:50, 50:50) **E_3_**: n_tot_= 800(100:100, 100:100, 100:100, 100:100)
	
4	**Within-center inequality:** **W_1_**: n_tot_= 160(10:30, 10:30, 10:30, 10:30) **W_2_**: n_tot_= 400(25:75, 25:75, 25:75, 25:75) **W_3_**: n_tot_= 800(50:150, 50:150, 50:150, 50:150)
	
	**Among-centers inequality:** **A_1_**: n_tot_= 160(10:10, 10:10, 10:10, 50:50) **A_2_**: n_tot_= 400(25:25, 25:25, 25:25, 125:125) **A_3_**: n_tot_= 800(50:50, 50:50, 50:50, 250:250)
	

	**Equal sample size design:** **E_1_**: n_tot_= 240(20:20, 20:20, 20:20, 20:20, 20:20, 20:20) **E_2_**: n_tot_= 600(50:50, 50:50, 50:50, 50:50, 50:50, 50:50) **E_3_**: n_tot_= 1200(100:100, 100:100, 100:100, 100:100, 100:100, 100:100)
	
6	**Within-center inequality:** **W_1_**: n_tot_= 240(10:30, 10:30, 10:30, 10:30, 10:30, 10:30) **W_2_**: n_tot_= 600(25:75, 25:75, 25:75, 25:75, 25:75, 25:75) **W_3_**: n_tot_= 1200(50:150, 50:150, 50:150, 50:150, 50:150, 50:150)
	
	**Among-centers inequality:** **A_1_**: n_tot_= 240(10:10, 10:10, 10:10, 10:10, 10:10, 70:70) **A_2_**: n_tot_= 600(25:25, 25:25, 25:25, 25:25, 25:25, 175:175) **A_3_**: n_tot_= 1200(50:50, 50:50, 50:50, 50:50, 50:50, 350:350)
	

	**Equal sample size design:** **E_1_**: n_tot_= 320(20:20, 20:20, 20:20, 20:20, 20:20, 20:20, 20:20, 20:20) **E_2_**: n_tot_= 800(50:50, 50:50, 50:50, 50:50, 50:50, 50:50, 50:50, 50:50) **E_3_**: n_tot_= 1600(100:100, 100:100, 100:100, 100:100, 100:100, 100:100, 100:100, 100:100)
	
8	**Within-center inequality:** **W_1_**: n_tot_= 320(10:30, 10:30, 10:30, 10:30, 10:30, 10:30, 10:30, 10:30) **W_2_**: n_tot_= 800(25:75, 25:75, 25:75, 25:75, 25:75, 25:75, 25:75, 25:75) **W_3_**: n_tot_= 1600(50:150, 50:150, 50:150, 50:150, 50:150, 50:150, 50:150, 50:150)
	
	**Among-centers inequality:** **A_1_**: n_tot_= 320(10:10, 10:10, 10:10, 10:10, 10:10, 10:10, 10:10, 90:90) **A_2_**: n_tot_= 800(25:25, 25:25, 25:25, 25:25, 25:25, 25:25, 25:25, 225:225) **A_3_**: n_tot_= 1600(50:50, 50:50, 50:50, 50:50, 50:50, 50:50, 50:50, 450:450)

Note: In the notation of E_i_: W_i_: and A_i_: n_tot_= n (n_11_: n_21_, ..., n_1k_: n_2k_): : is the total number of observations and *n_k _*is the number of observations per center.

## Results

### Equal sample size design

Tables 3 shows empirical type I error rate (when ) and power (when ) of the three homogeneity tests under the equal sample size design and various combinations of *n_k_*, *K*, , and . In terms of power, the tests are ordered BD≥DL≥LR in all cases and they perform similarly when the total sample size in each center is as large as 200 and . In addition, our findings reveal that substantial gain in the power of all homogeneity tests occurred with increasing number of centers, sample size per center and degree of heterogeneity of odds ratios, . However, increasing degree of heterogeneity of center, , from 0.1 to 0.5 resulted in a reduction of approximately 4.93% and 3.76% in the power of DL and BD, respectively, while no regular trend was detected for the change in LR.

**Table 3:** Type I error rate (when ) and the statistical power (when ) of homogeneity tests under equal sample size design.

															
			
		K = 4			K = 6			K = 8			K = 4			K = 6			K = 8		
							
	n_k_	LR	BD	DL	LR	BD	DL	LR	BD	DL	LR	BD	DL	LR	BD	DL	LR	BD	DL
**0**	**E_1_**	0.029	0.057	0.041	0.041	0.047	0.039	0.044	0.050	0.041	0.056	0.061	0.042	0.050	0.058	0.040	0.048	0.042	0.038
	**E_2_**	0.031	0.051	0.046	0.045	0.058	0.046	0.045	0.043	0.040	0.041	0.044	0.040	0.042	0.051	0.041	0.042	0.053	0.043
	**E_3_**	0.035	0.045	0.042	0.057	0.061	0.058	0.043	0.043	0.044	0.044	0.053	0.051	0.044	0.042	0.043	0.043	0.053	0.050

**0.2**	**E_1_**	0.134	0.220	0.183	0.167	0.316	0.256	0.215	0.365	0.287	0.155	0.207	0.151	0.219	0.277	0.209	0.244	0.336	0.266
	**E_2_**	0.396	0.470	0.459	0.450	0.590	0.570	0.569	0.718	0.699	0.401	0.467	0.452	0.460	0.560	0.545	0.561	0.653	0.638
	**E_3_**	0.594	0.697	0.692	0.739	0.841	0.836	0.862	0.917	0.915	0.609	0.668	0.661	0.772	0.807	0.804	0.840	0.890	0.881

**0.4**	**E_1_**	0.262	0.391	0.351	0.359	0.511	0.434	0.453	0.587	0.503	0.267	0.349	0.282	0.354	0.461	0.387	0.481	0.550	0.478
	**E_2_**	0.573	0.682	0.668	0.721	0.815	0.803	0.829	0.904	0.886	0.603	0.647	0.630	0.725	0.797	0.779	0.859	0.902	0.892
	**E_3_**	0.792	0.829	0.827	0.915	0.947	0.946	0.969	0.983	0.932	0.764	0.830	0.820	0.886	0.923	0.917	0.964	0.976	0.974

**0.6**	**E_1_**	0.367	0.482	0.427	0.518	0.659	0.607	0.634	0.756	0.673	0.368	0.468	0.401	0.515	0.600	0.517	0.628	0.701	0.642
	**E_2_**	0.674	0.780	0.765	0.833	0.875	0.862	0.927	0.965	0.960	0.692	0.750	0.718	0.839	0.891	0.877	0.918	0.943	0.929
	**E_3_**	0.844	0.882	0.879	0.963	0.979	0.978	0.989	0.992	0.992	0.835	0.869	0.862	0.939	0.956	0.953	0.983	0.996	0.996

**0.8**	**E_1_**	0.432	0.562	0.518	0.618	0.709	0.652	0.708	0.817	0.743	0.453	0.550	0.474	0.601	0.700	0.632	0.711	0.780	0.713
	**E_2_**	0.775	0.828	0.813	0.906	0.937	0.929	0.954	0.976	0.968	0.736	0.788	0.759	0.886	0.923	0.903	0.956	0.978	0.971
	**E_3_**	0.882	0.919	0.918	0.973	0.987	0.987	0.996	0.997	0.997	0.883	0.902	0.896	0.969	0.980	0.975	0.992	0.996	0.996

The notation of E_i _is described in Table 2

### Unequal sample size designs

Table 4 shows type I error rate and the power of the homogeneity tests under within-center inequality for different values of *n_k_*, *K*, , and . Indeed, when  = 0.1, as compared with the equal sample size design, there were decreases of approximately 16.35%, 9.93% and 11.62% in the power of LR test for within-center inequality, when *K *was equal to 4, 6 and 8, respectively. In addition, the power of the BD statistic decreased approximately by 10.67%, 7.79% and 7.15% and the power of DL was reduced approximately by 14.46%, 11.24% and 9.76% for *K *= 4, 6 and 8, respectively. It should be noted that, for a given value of *K*, the reported amount of reduction was the average reduction for all values of .

**Table 4:** Type I error rate (when ) and the statistical power (when ) of homogeneity tests under within-center inequality.

															
			
		K = 4			K = 6			K = 8			K = 4			K = 6			K = 8		
							
	n_k_	LR	BD	DL	LR	BD	DL	LR	BD	DL	LR	BD	DL	LR	BD	DL	LR	BD	DL
**0**	**W_1_**	0.039	0.057	0.037	0.047	0.064	0.041	0.059	0.064	0.041	0.041	0.045	0.039	0.039	0.042	0.039	0.042	0.043	0.034
	**W_2_**	0.035	0.041	0.041	0.045	0.041	0.045	0.043	0.053	0.044	0.035	0.060	0.048	0.041	0.044	0.040	0.039	0.043	0.045
	**W_3_**	0.037	0.044	0.044	0.046	0.055	0.050	0.041	0.057	0.052	0.032	0.043	0.040	0.041	0.058	0.053	0.041	0.055	0.049

**0.2**	**W_1_**	0.091	0.185	0.129	0.177	0.275	0.198	0.154	0.292	0.209	0.127	0.175	0.118	0.133	0.200	0.117	0.173	0.254	0.159
	**W_2_**	0.271	0.395	0.394	0.412	0.531	0.501	0.474	0.626	0.596	0.282	0.392	0.360	0.381	0.499	0.455	0.477	0.585	0.533
	**W_3_**	0.501	0.629	0.621	0.695	0.757	0.755	0.758	0.840	0.835	0.517	0.572	0.559	0.678	0.742	0.728	0.759	0.838	0.828

**0.4**	**W_1_**	0.191	0.305	0.221	0.279	0.417	0.323	0.341	0.518	0.371	0.236	0.294	0.219	0.269	0.364	0.250	0.372	0.481	0.436
	**W_2_**	0.466	0.593	0.580	0.655	0.768	0.746	0.742	0.856	0.832	0.452	0.558	0.530	0.643	0.723	0.676	0.774	0.844	0.811
	**W_3_**	0.723	0.807	0.802	0.880	0.918	0.914	0.939	0.969	0.965	0.716	0.784	0.775	0.887	0.910	0.906	0.949	0.966	0.965

**0.6**	**W_1_**	0.294	0.430	0.352	0.399	0.574	0.469	0.494	0.652	0.537	0.286	0.399	0.318	0.387	0.497	0.391	0.484	0.599	0.468
	**W_2_**	0.615	0.703	0.682	0.780	0.853	0.828	0.862	0.918	0.906	0.599	0.686	0.648	0.771	0.830	0.799	0.895	0.919	0.894
	**W_3_**	0.817	0.858	0.855	0.949	0.957	0.957	0.976	0.987	0.985	0.795	0.845	0.832	0.926	0.951	0.941	0.968	0.981	0.977

**0.8**	**W_1_**	0.356	0.471	0.398	0.530	0.647	0.540	0.641	0.767	0.663	0.363	0.459	0.362	0.516	0.608	0.514	0.631	0.715	0.594
	**W_2_**	0.687	0.769	0.742	0.837	0.888	0.868	0.915	0.969	0.966	0.694	0.751	0.701	0.835	0.887	0.843	0.922	0.944	0.915
	**W_3_**	0.857	0.891	0.889	0.957	0.970	0.968	0.990	0.995	0.993	0.845	0.874	0.861	0.955	0.966	0.963	0.982	0.984	0.983

The notation of W_i _is described in Table 2

Type I error rate and the power of the homogeneity tests under among-centers inequality are illustrated in Table 5. In fact, when  = 0.1 as compared with the equal sample size design, there were decreases of approximately 17.15%, 11.78% and 11.89% in the power of LR test and 16.63%, 13.62% and 12.57% in the power of the BD statistic for among-centers inequality, when *K *was equal to 4, 6 and 8, respectively. In addition, this amount of reduction in the power of DL test was more critical and approximately equal to 21.56%, 18.4% and 16.61% for the same sequence of *K*.

**Table 5:** Type I error rate (when ) and the statistical power (when ) of homogeneity tests under among-centers inequality.

															
			
		K = 4			K = 6			K = 8			K = 4			K = 6			K = 8		
							
	n_k_	LR	BD	DL	LR	BD	DL	LR	BD	DL	LR	BD	DL	LR	BD	DL	LR	BD	DL
**0**	**A_1_**	0.030	0.045	0.041	0.045	0.045	0.037	0.041	0.041	0.036	0.031	0.041	0.036	0.045	0.041	0.038	0.044	0.040	0.036
	**A_2_**	0.031	0.047	0.043	0.048	0.046	0.039	0.045	0.056	0.043	0.037	0.053	0.042	0.039	0.049	0.040	0.039	0.044	0.040
	**A_3_**	0.033	0.057	0.054	0.043	0.045	0.041	0.046	0.042	0.045	0.039	0.055	0.044	0.041	0.047	0.042	0.049	0.042	0.041

**0.2**	**A_1_**	0.121	0.168	0.120	0.176	0.225	0.161	0.215	0.259	0.168	0.105	0.149	0.108	0.182	0.165	0.090	0.248	0.236	0.131
	**A_2_**	0.265	0.362	0.334	0.395	0.479	0.440	0.472	0.583	0.534	0.281	0.343	0.309	0.378	0.458	0.407	0.459	0.508	0.451
	**A_3_**	0.512	0.602	0.595	0.645	0.749	0.739	0.723	0.817	0.805	0.492	0.577	0.560	0.699	0.701	0.676	0.710	0.795	0.767

**0.4**	**A_1_**	0.187	0.262	0.194	0.312	0.397	0.306	0.359	0.469	0.352	0.251	0.299	0.244	0.339	0.360	0.248	0.369	0.385	0.254
	**A_2_**	0.437	0.555	0.518	0.587	0.707	0.658	0.720	0.814	0.765	0.457	0.550	0.501	0.601	0.687	0.617	0.692	0.796	0.721
	**A_3_**	0.676	0.754	0.744	0.838	0.896	0.885	0.884	0.941	0.934	0.686	0.744	0.728	0.802	0.871	0.853	0.902	0.929	0.924

**0.6**	**A_1_**	0.292	0.367	0.289	0.397	0.493	0.390	0.493	0.564	0.457	0.281	0.346	0.272	0.402	0.469	0.353	0.538	0.562	0.421
	**A_2_**	0.602	0.692	0.651	0.720	0.810	0.762	0.831	0.906	0.874	0.574	0.631	0.575	0.726	0.809	0.745	0.791	0.856	0.797
	**A_3_**	0.755	0.824	0.811	0.909	0.937	0.929	0.937	0.972	0.969	0.740	0.801	0.784	0.902	0.935	0.917	0.959	0.971	0.960

**0.8**	**A_1_**	0.345	0.430	0.355	0.466	0.586	0.473	0.580	0.668	0.551	0.384	0.430	0.356	0.490	0.547	0.459	0.605	0.631	0.492
	**A_2_**	0.670	0.755	0.710	0.816	0.878	0.829	0.870	0.933	0.889	0.644	0.729	0.664	0.805	0.868	0.806	0.868	0.926	0.869
	**A_3_**	0.817	0.867	0.856	0.930	0.952	0.940	0.973	0.980	0.979	0.819	0.850	0.836	0.928	0.952	0.942	0.966	0.990	0.981

The notation of A_i _is described in Table 2

It should be pointed out that the amount of reduction in the power of the three homogeneity tests for  = 0.5 under two forms of unequal sample size designs was approximately similar to those of  = 0.1.

### Type I error rate

Tables 3, 4 and 5, when  = 0, represent empirical type I error rate of the three homogeneity tests at the nominal significance level of 0.05. As indicated, LR test showed conservative behavior when *K *= 4, otherwise, the type I error rate was close to the nominal level. In addition, BD performed adequately in terms of type I error rate in almost all cases. On the other hand, type I error rate of the DL statistic was close or below the nominal level.

## Discussion and conclusions

In a simple randomized clinical trial, the use of unequal allocation ratios, particularly the allocation ratio of 3:1, will significantly reduce the power of study for detecting significance difference between two treatments [13-15]. To our knowledge, few published studies investigated the impact of within-center and among-centers inequality on the statistical properties of the tests of homogeneity of odds ratios in multicenter clinical trials [1,3,4]. As illustrated in Tables 3, 4 and 5, the type I error rate of the three homogeneity tests is approximately close to the nominal level of 0.05 except for LR when *K *= 4. Since the results show that these tests have almost the same type I error rate, power comparisons are possible. As compared with the equal sample size design, the power of the LR, BD and DL tests will decrease if the same total sample size, which can be allocated equally within one center or among centers, is allocated unequally. In this case, the power ranking of the tests was BD≥DL≥LR. It is worth mentioning that, as compared with within-center inequality, among-centers inequality has stronger adverse effect on the power of the homogeneity tests. Despite the use of different tests, these findings are inconsistent with those of Paul, who reported the adverse effect of within-center inequality to be stronger [3].

Also, this paper shows how to use a mixed logistic model to test homogeneity of odds ratios in multicenter trials. In Model 1, there are two types of homogeneity: homogeneity of odds ratios among centers and homogeneity of centers. However, removing the center-by-treatment interaction from Model 1 leads to a model which can only be used to test homogeneity of centers. This model, which has been previously discussed by Gao, assumes that the odds ratios are constant over centers [16]. Therefore, it should not be used to generate data for comparing the tests of heterogeneity of odds ratios. Furthermore, the power of the three tests of homogeneity increases more when we increase the number of *K *and *n_k _*compared to when we increase the number of *K *and . This result is in agreement with previous studies which have evaluated the influence of *K *and *n_k _*on the power of the homogeneity tests [5,17-19]. Nevertheless, our simulation study shows that the degree of among-centers heterogeneity, , has little or no effect on the power of the three tests of homogeneity, except for DL when  and sample size is small.

In addition, it is noteworthy that we used the DL statistic calculated from the one-way random effects model, which has approximately a chi-square distribution. However, Biggerstaff and Jackson [20] have calculated the exact distribution and power of the well-known Q statistic based on the same random effects model, which can be used for testing homogeneity of odds ratios and be compared with the tests used in the present study.

In conclusion, of the three tests of homogeneity, the BD seems to be the most appealing with regard to its statistical properties: its type I error rate is close to the nominal level and its power is greater than that of DL and LR. Moreover, it has the advantage of simplicity of calculation and is recommended by a number of authors [1,4-6]. However, one limitation of BD test is that it has low power when the sample size within each center is small, even if the number of centers is large [1,2]. Nevertheless, despite having low power under small number of centers and its complexity, Model 1 has its own advantages. Firstly, when the centers are a random sample themselves, the LR test from the Model 1 enables inferences to extend to the population of centers. Secondly, a further consideration is that common odds ratio can be estimated from the fixed part of the Model 1, even when the odds ratios are not homogeneous. Thirdly, in each center, Model 1 provides a predicted log-odds ratio that shrinks the sample value toward the mean. This is especially useful when the sample size in a center is small and the ordinary sample odds ratio has a large standard error [8]. In addition, the mixed logistic model described in this study will potentially be applicable to meta-analysis studies.

It is clear that, based on Model 1, the odds ratio in the *k*th center, as given in the appendix 4, is *exp(β + b_1k _*- *b_2k_)*, which can be written as *C × exp(b_1k _*- *b_2k_) *where *C = exp(β) *.This indicates that the odds ratio in each center is absolutely independent of *α *and *u_k_*. Indeed, the odds ratios are affected by *b*_1*k *_and *b*_2*k*_, and *β *has the same effect on odds ratio in all centers. Hence, to generate heterogeneous odds ratios among centers, the fixed simulation parameters, ie *α *and *β*, can be chosen arbitrarily.

It should be noted that, although using unequal sample size designs in multicenter clinical trials reduces both the power of the study and the power of the homogeneity tests, a substantial reduction in the total cost of the trial will compensate for the reduction in the power of the statistical tests [14,15]. Finally, further research is warranted to investigate the influence of the number of centers, unequal sample size design, sparseness and also deviation from normal assumption of the random effects on the robustness and accuracy of the estimates of the fixed and random parameters of the Model 1.

## Appendix

### 1. Breslow-Day statistic

Breslow and Day [9] proposed the test statistic:

Where *OR_MH _*is the Mantel-Haenszel estimator of common odds ratio. *E(y_1k_*|*OR_MH_) *and *V(y_1k_|OR_MH_) *are the expected value and variance of *y*_1*k *_under the null hypothesis of homogeneity of odds ratios. Under the assumption of large sample size in each 2 × 2 table, BD has approximately chi-square distribution with *k *- 1 degree of freedom [9].

### 2. DerSimonian-Laird statistic

The Dersimonian and laird statistic is based on random effects model, which obtained by:

When log-odds ratio is used as a summary measure ie, common treatment effect, *y_k _= ln(OR_k_) *where *ln(OR_k_) *is the log-odds ratio in *k*th center and  where . DL statistic has approximately chi-square distribution with *k *- 1 degree of freedom [10].

### 3. Likelihood ratio test based on mixed logistic models

To test homogeneity of odds ratios based on mixed logistic models, we compare the model

to the simpler model not containing the random effect term of the center-by-treatment interaction, namely:

which is equivalent to test the null hypothesis of  versus [8]. As the null hypothesis is on the boundary of the parameter space, the asymptotic null distribution for the likelihood ratio test is a mixture of a  and a  with equal probability 1/2, rather than classical single chi-square distribution [11,12].

### 4. Calculation of odds ratio based on the mixed logistic model with two random effects

Based on Model * which was presented above, the odds of success in treatment 1 in *kth *center is derived as:

and in a similar manner the odds of success in treatment 2 is derived as:  therefore, the odds ratio for treatment 1 versus 2 in kth center is *exp(β+b_1k _- b_2k_)*.

### 5. SAS code for performing likelihood ratio test in a 2 × 2 × 2 contingency tables

data binomial;

input center treat y n; * y successes out of n trials;

cards;

1 1 30 100

1 2 50 100

2 1 45 100

2 2 75 100

;

run;

proc nlmixed data = binomial qpoints = 15; *Mixed logistic model with one random effect, *no interaction;*

parms alpha = -1 beta = 1 su = 0.2; *Initial values for parameters estimates;

bounds su>=0;

z=alpha+beta*treat+u; *Logistic formula;

expz=exp(z);

pi=expz/(1+expz);

model y~binomial(n,pi);

random u~ normal(0,su*su) subject=center;

ods output FitStatistics=test1;

ods listing select test1;

run;

proc nlmixed data=binomial qpoints = 15; *Mixed logistic model with two random effects, interaction;

parms alpha=-1 beta = 1 su = 0.2 sb = 0.8; *Initial values;

bounds su>0; bounds sb>0;

z=alpha+beta*treat+a+b*treat; *Logistic formula;

expz=exp(z);

pi=expz/(1+expz);

model y~binomial(n,pi);

random a b~ normal([0,0],[su*su,0,sb*sb]) subject=center;

ods output FitStatistics=test2;

ods listing select test2;

run;

data mixed; *Calculating likelihood ratio statistic;

merge test1(rename=(value=d1)) test2(rename=(value=d2));

if descr='-2 Log Likelihood';

run;

data combmix; set mixed;

delta=d1-d2;

run;

data lr; set combmix;

x=probchi(delta,1); *Testing hypothesis of homogeneity of odds ratios, based on mixture chi-square;

run;

data rejectmixed;

set lr;

rej1 = 0.5*(1-x);

a=(rej1<0.05);

run;

## Competing interests

The authors declare that they have no competing interests.

## Authors' contributions

ZB and PJ were responsible for the design, simulation, analysis and interpretation. SMTA supervised the study and interpreted the results. All authors read and approved the final manuscript.

## Pre-publication history

The pre-publication history for this paper can be accessed here:

http://www.biomedcentral.com/1471-2288/11/58/prepub

## Supplementary Material

Additional file 1**SAS simulation code for performing the three homogeneity tests of odds ratios in a certain case, in which K = 6, n_ik _= 100,  and **.Click here for file

## References

[B1] Jones MP O'Gorman TW Lemke JH Woolson RF A Monte Carlo investigation of homogeneity tests of the odds ratio under various sample size configurations Biometrics 1989 45 171 81 10.2307/2532043 2720050

[B2] O'Gorman TW Woolson RF Jones MP Lemke JH Statistical analysis of K 2 × 2 tables: A comparative study of estimators/test statistics for association and homogeneity Environ Health Perspect 1990 87 103 107 2269213 10.1289/ehp.9087103 PMC1567816

[B3] Paul SR Donner A A comparison of tests of homogeneity of odds ratios in K 2 × 2 tables Stat Med 1989 8 1455 1468 10.1002/sim.4780081205 2616935

[B4] Reis IM Hirji KF Afifi AA Exact and asymptotic tests for homogeneity in several 2 × 2 tables Stat Med 1999 18 893 906 10.1002/(SICI)1097-0258(19990430)18:8<893::AID-SIM84>3.0.CO;2-5 10363329

[B5] Gavaghana DJ Moore RA McQuay HJ An evaluation of homogeneity tests in meta-analyses in pain using simulations of individual patient data Pain 2000 85 415 424 10.1016/S0304-3959(99)00302-4 10781914

[B6] Paul SR Donner A Small sample performance of tests of homogeneity of odds ratios in K 2 × 2 tables Stat Med 1992 11 159 165 10.1002/sim.4780110203 1579755

[B7] Mittlböck M Heinzl H A simulation study comparing properties of heterogeneity measures in meta-analyses Stat Med 2006 25 4321 4333 10.1002/sim.2692 16991104

[B8] Agresti A Hartzel J Strategies for comparing treatments on a binary response with multi-centre data Stat Med 2000 19 1115 1139 10.1002/(SICI)1097-0258(20000430)19:8<1115::AID-SIM408>3.0.CO;2-X 10790684

[B9] Breslow N Day NE Calssical methods of analysis of grouped data Statistical Methods in Cancer Research. The Analysis of Case-Control Studies 1980 1 Lyon: International Agency for Research on Cancer 122 159

[B10] DerSimonian R Laird N Meta-analysis in clinical trials Control Clin Trials 1986 7 177 188 10.1016/0197-2456(86)90046-2 3802833

[B11] Verbeke G Molenberghs G The use of score tests for inference on variance components Biometrics 2003 59 254 262 10.1111/1541-0420.00032 12926710

[B12] Molenberghs G Verbeke G Likelihood ratio, score, and Wald tests in a constrained parameter space Am Statistician 2007 61 1 6 10.1198/000313007X168173

[B13] Jafari P Ayatollahi SMT Behboodian J Sequential boundaries approach in clinical trials with unequal allocation ratios BMC Med Res Methodol 2006 6 1 10.1186/1471-2288-6-1 16412232 PMC1382245

[B14] Torgerson DJ Campbell MK Use of unequal randomisation to aid the economic efficiency of clinical trials BMJ 2000 321 759 10.1136/bmj.321.7263.759 10999916 PMC1127870

[B15] Torgerson DJ Campbell MK Unequal randomisation can improve economic efficiency of clinical trials J Health Serv Res Policy 1997 2 81 85 10180369 10.1177/135581969700200205

[B16] Gao S Combining binomial data using the logistic normal model J Stat Comput 2004 74 293 306 10.1080/0094965031000151169

[B17] Hardy RJ Thompson SG Detecting and describing heterogeneity in meta-analysis Stat Med 1998 17 841 856 10.1002/(SICI)1097-0258(19980430)17:8<841::AID-SIM781>3.0.CO;2-D 9595615

[B18] Huedo-Medina TB Sanchez-Meca J Marin-Martinez F Assessing heterogeneity in meta-analysis: Q statistic or I^2 ^index? Psychol Methods 2006 11 193 206 16784338 10.1037/1082-989X.11.2.193

[B19] Viechtbauer W Hypothesis tests for population heterogeneity in meta-analysis Br J Math Stat Psychol 2007 60 29 60 10.1348/000711005X64042 17535578

[B20] Biggerstaff BJ Jackson D The exact distribution of Cochran's heterogeneity statistic in one-way random effects meta-analysis Stat Med 2008 27 6093 6110 10.1002/sim.3428 18781561

